# International validation of the EORTC QLQ-PRT20 module for assessment of quality of life symptoms relating to radiation proctitis: a phase IV study

**DOI:** 10.1186/s13014-018-1107-x

**Published:** 2018-08-29

**Authors:** Georgia K. B. Halkett, Charles Adam Wigley, Samar M Aoun, Maurizio Portaluri, Francesco Tramacere, Lorenzo Livi, Beatrice Detti, Stefano Arcangeli, Jo-Asmund Lund, Are Kristensen, Nathalie McFadden, Arne Grun, Sean Bydder, Irina Sackerer, Elfriede Greimel, Nigel Spry, on behalf of the EORTC Quality of Life Group, Celine Fournier, Melissa Berg, Myriam Bouchard, Anne-Sophie Lessard, Julie Villa

**Affiliations:** 1 School of Nursing, Midwifery and Paramedicine, Curtin University, GPO Box U1987, Perth, WA 6845 Australia; 2 Institute for Health Research, University of Notre Dame, Perth, Western Australia; 3 Palliative Care Unit, School of Psychology and Public Health, La Trobe University, Bundoora, VIC Australia; 4 Radiation Oncology Dept. “A. Perrino” General Hospital, ASL Brindisi, Italy; 5 University of Florence, Florence, Italy; 6 San Camillo and Forlanini Hospitals, Rome, Italy; 7 Cancer Clinic, St. Olavs University Hospital, Trondheim, Norway; 8 Centre Hospitalier Universitaire de Sherbrooke, Sherbrooke, Quebec Canada; 9 Charité - University Medicine, Berlin, Germany; 10 Department of Radiation Oncology, Sir Charles Gairdner Hospital, Perth, Western Australia; 11 Department of Radiation Oncology, Technical University of Munich, Munich, Germany; 12 Radiation Oncology, Freising and Dachau, Germany; 13 Medical University Graz, Graz, Austria; 14 Genesis Cancer Care, Joondalup, WA Australia

## Abstract

**Background:**

Although patients experience radiation proctitis post radiotherapy no internationally tested instruments exist to measure these symptoms. This Phase IV study tested the scale structure, reliability and validity and cross-cultural applicability of the EORTC proctitis module (QLQ-PRT23) in patients who were receiving pelvic radiotherapy.

**Methods:**

Patients (*n* = 358) from six countries completed the EORTC QLQ-C30, QLQ-PRT23 and EORTC Quality of Life Group debriefing questions. Clinicians completed the EORTC Radiation Therapy Oncology Group scale. Questionnaires were completed at four time-points. The module’s scale structure was examined and validated using standard psychometric analysis techniques.

**Results:**

Three items were dropped from the module (QLQ-PRT23 → QLQ-PRT20). Factor analysis identified five factors in the module: bowel control; bloating and gas; emotional function/lifestyle; pain; and leakage. Inter-item correlations were within *r* = 0.3–0.7. Test-Retest reliability was high. All multi-item scales discriminated between patients showing symptoms and those without symptomology. The module discriminated symptoms from the clinician completed scoring and for age, gender and comorbidities.

**Conclusion:**

The EORTC QLQ-PRT20 is designed to be used in addition to the EORTC QLQ-C30 to measure quality of life in patients who receive pelvic radiotherapy. The EORTC QLQ-PRT20 is quick to complete, acceptable to patients, has good content validity and high reliability.

**Trial registration:**

Australian and New Zealand Clinical Trials Registry (ANZCTR) ACTRN12609000972224.

**Electronic supplementary material:**

The online version of this article (10.1186/s13014-018-1107-x) contains supplementary material, which is available to authorized users.

## Background

‘Radiation proctitis’ or ‘pelvic radiation disease’ is an unpleasant recurrent symptom that occurs following pelvic radiotherapy. Patients present with: anorectal pain, rectal bleeding and/or blood clots, bowel urgency, frequent diarrhoea, profuse mucous discharge, faecal and/or mucous incontinence [1–4]. Symptoms can have profound social and psychological consequences for patients and their families [5, 6].

The reported incidence of radiation proctitis ranges between 2 and 20% [2, 7]. However, the true incidence is likely to have been underestimated as clinician evaluation of symptoms utilise toxicity scales which focus on rectal bleeding and do not include assessment of urgency of defaecation and/or mucous/faecal incontinence. The move to conformal radiation techniques and intensity modulated radiotherapy has helped to reduce toxicity [8–10]. Conversely, there has been a trend towards dose escalation studies [11], external beam radiotherapy plus high dose rate brachytherapy [12] and an increase in the range of indications for pelvic radiation [13]. Prospective trials are needed to establish the true incidence of the condition, the effect it has on patients’ quality of life (QoL) and the best forms of treatment.

QoL instruments provide a reliable and valid method of assessing the impact of treatment on patients’ lives and evaluating topical, medical, nutritional and surgical options [14]. A recent systematic review provides a summary of the most frequently used QoL questionnaires for prostate cancer [15]. The EORTC Quality of Life Group (QLG) modules developed for cervical (CX24) and prostate (PR25) cancer are also useful for identifying disease-specific issues, but do not adequately address problems associated with radiation proctitis. The one questionnaire directly assessing QoL in patients with radiation proctitis fails to address all the proctitis related issues and has not been validated internationally [5]. The EORTC QLG therefore developed and tested a module specifically for radiation proctitis that could be administered with the EORTC QLQ-C30 (EORTC core questionnaire for quality of life) [16]. We have published Phase I-III testing of the EORTC proctitis module in Australia [17] and Phase III pretesting of the module internationally [18].

The primary objective of this Phase IV study was to test the scale structure, reliability, validity and cross-cultural applicability of the proctitis module in patients who were receiving pelvic radiotherapy.

## Methods

This prospective multi-centre study followed the EORTC QLG guidelines for module development [19]. QoL data was collected alongside socio-demographic and clinical background data (including comorbidities). A trial protocol was developed and reviewed by the EORTC QLG prior to commencing the study. Ethics approval was gained from Curtin University and participating sites. The trial was also registered on the Australian and New Zealand Clinical Trials Registry (ANZCTR) ACTRN12609000972224.

### Participants

Patients were recruited from Australia, Italy, Norway, Canada (French speaking), France and Germany. This enabled testing of the module in English, Norwegian, Italian, German and French. The module was translated for each language in collaboration with the translation team at the EORTC Quality of Life Department [20].

Patients were eligible if they were receiving a radical course of pelvic irradiation (> 45 Gray) and were able to converse freely in the language that the questionnaire was written. Patients were ineligible if they had previously received radiotherapy or the radiation dose prescribed was less than 45 Gray.

### Data collection

Radiation oncologists identified patients eligible for the study. Written informed consent was obtained. Participants were recruited from March 2010 and data collection ceased May 2014.

Data was collected at the following time-points:

1. At least 2 weeks prior to radiotherapy treatment (when they saw their radiation oncologist) (T1);
2. During first week of treatment (T2);
3. End of treatment (T3);
4. At the three to 6 months scheduled follow-up appointment, after treatment completion (T4).

Patients completed the first round questionnaires when they saw their radiation oncologist prior to commencing treatment. On subsequent occasions the questionnaires were either provided when they were attending for treatment or posted to them.

Patients completed a demographics questionnaire, the EORTC QLQ-C30 [16] and the proctitis module (QLQ-PRT23). They also responded to these debriefing questions:

- How long did it take you to complete the questionnaire?
- Did anyone help you to complete the questionnaire?
- Were there questions that you found confusing or difficult to answer?
- Were there questions that you found upsetting?

The treating radiation oncologists completed the EORTC Radiation Therapy Oncology Group (RTOG) classification system at each time-point [21].

### Recruitment targets

The primary endpoint was to evaluate the scale structure of the QLQ-PRT23. Using the EORTC QLG guidelines for sample size calculation the accepted ‘rule of thumb’ is that 15 responses per item are needed [22]. As the Phase III module had 21 items, and allowing for a 10% dropout rate, we needed a sample of 350 participants.

### Statistical analysis

The original study design was based on the 2002 EORTC QLG guidelines for module development [19]. The statistical analysis plan was updated to reflect the 2011 EORTC QLG guidelines for module development(Version 4) [22]. All analyses were conducted using SPSS version 23 with alpha levels set to *p* < .05.

#### Scale structure

The initial stage of the development of the EORTC proctitis module identified seven areas of interest for providing QoL information specific to radiation proctitis: incontinence, pain, bleeding, social function, role and performance, fatigue and emotional function. Of these, fatigue was dropped after consultation with health professionals and patients (Phase 1b). While the question items in subsequent versions of the proctitis module (PRT21, PRT23) were based on the remaining six areas, this paper presents the first formal analysis of the scale structure of this module.

Because radiation proctitis occurs as a possible side effect of pelvic radiotherapy the exploration of the scale structure of the module was performed on T3 (post treatment completion) data.

Exploratory Factor Analysis (EFA) was used to investigate the structure of QLQ-PRT23 questions 31–51 [23]. Questions 52–54 were not included in the item and scale structure review analysis because they were single-item scales and collected specific clinical information relevant to patient comfort and future treatment.

The results of the EFA were refined through consultation with the research team. Multi-trait scaling analysis was used to test the construct validity (convergent and discriminant validity) of the proposed multi-item scales. Within the module, convergent validity was considered adequate when an item was highly correlated to its own proposed scale corrected for overlap, operationally defined as *r* ≥ 0.4 [23]. Discriminant validity was supported when an item demonstrated lower correlations with other proposed scales compared to its own proposed scale. Possible scaling errors were flagged when an item correlated more highly to some other scale compared to its own proposed scale [16]. Scaling failures were identified when this difference was greater than two standard errors [23].

#### Reliability

The homogeneity, usefulness and level of fit of items within the proposed scale were examined. The internal reliability for the scales was assessed using Cronbach’s alpha coefficient with an *r* ≥ 0.70 considered adequate^27^.

Intra-class correlations coefficients (ICC) of the proposed scales between T1 (2 weeks before treatment) and T2 (first week of treatment) data sets and their test re-test reliabilities were examined. Significant differences in patient responses were not expected at these time-points [24]. ICC’s of > 0.7 were considered acceptable and ICC’s > 0.9 were excellent [25].

#### Validity

Convergent validity of the QLQ-PRT20 was examined via correlations with conceptually similar rating scales from the QLQ-C30. Based on the item names, previous literature and team discussion prior to analysis the following scales were expected to correlate highly (*r* ≥ 0.4) at T3: QLQ-PRT20 Emotional Function/Lifestyle scale and the QLQ-C30 Global Health Status and Emotional and Social scales; QLQ-PRT20 Pain scale and the QLQ-C30 Pain scale.

Two methods were used to assess discriminant validity: Known Groups Analysis based on age [26], location/type of tumour, and presence of co-morbidities [27], and Responsiveness to Change at T2 (first week of treatment) and T3 (Post treatment).

## Results

In total, 358 patients participated: 181 from Australia; 86 from Italy; 47 from Norway; 34 from Canada (French speaking); four from France; and six from Germany. Patient demographics and clinical characteristics by country are summarized in Table 1. Further details about the treatment patients received for each diagnosis are provided in Additional file 1 and patient comorbidities are shown in Additional file 2. The attrition rate was 10%.

**Table 1:** Patient characteristics at study commencement

Patient characteristics	Australia	Italy	Norway	French Speaking		Germany	Totals
				Canada	France		
Number of patients	181	86	47	34	4	6	358
Mean age in years (SD)	69.2 (9.3)	63.7 (10.5)	68.5 (5.6)	69.5 (6.0)	62.5 (9.3)	69.7 (8.2)	67.7 (9.2)
Range in years	25–95	39–82	55–78	56–80	49–70	55–79	25–95
Male %	96.7	64	97.9	100	50	100	88.9
Patients with one or more existing co-morbidities	105	32	27	26	3	3	196
Site							
Anal Canal		3					3
Bladder	2	2					4
Cervix		18					19
Chordoma				1			1
Endometrium		10					10
Iliac Nodes	1						1
Prostate	162	44	44	28	2	6	286
Rectum	15	8	3	5	1		32
Sigmoid Colon	1				1		2

NB: to gain power the cultural applicably analysis was conducted by language group. To achieve this participants from France and Canada were combined into the French speaking cohorts and the 6 German participants were excluded due to lack of numbers. Data from the German participants was included in the broader analysis

### Qualitative feedback

Qualitative survey responses were received at each time-point. At T1 responses were received from 281 participants. The response rates for follow-up questionnaires were similar (80%). Table 2 summarises response patterns for the follow-up questionnaires. Overall, 62% of participants who completed the qualitative follow up questions at T1 indicated they completed the questionnaires within 10 min, 23.5% took 11–15 min and 11.4% took 16–20 min. Similar rates were reported across the other times points.

**Table 2:** Response patterns for qualitative follow up questions

Time	Valid response N’s to items: Median (range)	Questions								
		Time to complete			Received Help		Confusing questions		Upsetting questions	
		< 10 min	10–15 Min	> 15 Min	No	Yes	No	Yes**	No	Yes
T1	261 (254–281)	175	66	40	220	34 (30 = family, 4 health professionals)	228	34 (Q42 *n* = 6; Q43 *n* = 3; Q51 n = 3; Specific to bowel questions *n* = 8; other questions *n* = 2 or less)	253	6 (Q51 *n* = 2, all others singletons)
T2	249 (242–265)	174	60	31	220	22(15 explicitly ref., friends or family, others no additional info)	222	25 (Uninformative affirmatives *n* = 6; C30 questions *n* = 4; questioning bowel questions *n* = 3; Q41 n = 3; Q 51 *n* = 3)	243	2 (Q51)
T3	243 (240–255)	154	79	30	213	26 (13 family, 3 health professionals, others no additional info)	220	25 (Uninformative affirmatives n = 6; C30 questions *n* = 5; questioning urinary issues *n* = 2; Q41 *n* = 2; Q51 *n* = 3)	238	5 (Singletons on Q32, 42, 43 & 51, uninformative affirmative *n* = 1)
T4	233 (229–245)	150	69	26	214	15 (10 family; others no additional info)	225	4 (Q51 *n* = 2; others singletons for Q49, 52, 54)	225	4 (Q51 *n* = 2; singletons for Q 49, 50, 52 & 54)

Thirteen percent found at least one question confusing at T1, but most of these were related to bowel issues. Few issues were reported with the bowel related questions during or post treatment. Overall, the majority of participants did not have problems understanding the questions. Of those respondents who provided written comments: most provided additional clarifications and/or elaborated on co-morbidity issues.

### Completion of QLQ questionnaires

The numbers of patients returning completed QLQ questionnaires (QLQ-C30 and QLQ-PRT23) was approximately 98% at T1. For the QLQ-PRT23 missing item rates were approximately 1%. Question 51 (Q51) “How unhappy would you feel if you lived the rest of your life with your bowel habit as it is now?” recorded the highest missing data (3.7% at T1). Participants may not have answered this question at T1 because they had not commenced radiotherapy. The question with the highest and most consistent rate of missing entries was Q53 “Highest number of times you had to open your bowels in any 24 hour period” with rates between 3 and 4% across T1-T4.

### Cultural applicability

Investigation of possible cultural factors influencing the completion of the QLQ-PRT23 focused on the four main language groups rather than patient’s country of treatment: English, Italian, Norwegian and French (see Table 1).

Comparing these language groups, 78% of women recruited were from the Italian cohort. The Italian cohort were 2.8 times *less* likely (odds ratio) to have a comorbid disorder than members of the other language groups while the French cohort were 2.95 times *more* likely; *χ*2(3) = 18.73, *p* < .001. The Italian cohort were also significantly younger (5 years) than the other language groups; *F*(3,348) = 8.03, *p* < .001.

The response patterns for the T3 QLQ-PRT23 was found to be similar across the language groups although the Italian and Norwegian cohorts had higher symptom reporting on some items compared to the French and English-speaking cohorts: Specifically, items with higher symptom reporting for the Norwegian cohort were Q32, 42, 43 and high scores for the Italian cohort were Q40 and 48.

#### Item and scale structure review

Item and scale structure were assessed using the T3 QLQ-PRT23 responses. The QLQ-PRT23 consisted of 23 items with one optional question. The final three questions (Q52–54) were not included in the item and scale structure review as they concerned specific clinical information.

After review, items Q41 (presence of dark blood in stools), Q45 (Have you had to wear a pad because of your bowel problems?) and Q47 (Have your daily activities been limited by your bowel problems?) were dropped due to low prevalence, poor fit and/or multicollinearity.

The final module analysed was 20 items with an optional additional question and will now be identified as EORTC QLQ-PRT20.

Numerous factor structures were explored starting with the original six areas of interest which showed relatively poor fit. A five-factor structure best accounted for the data and after team discussions the structure shown in Table 3 was chosen as the most clinically parsimonious. Five factors descriptors were assigned: Bowel Control; Bloating and Gas; Emotional Function/Lifestyle; Pain and Leakage.

**Table 3:** Scale structure for the proposed EORTC QLQ PRT20 module

Scale	Question
Bloating and Gas	Q31. Have you had a bloated feeling in your abdomen?
Bloating and Gas	Q32. Were you troubled by passing wind / gas / flatulence?
Bloating and Gas	Q33. Have you had excessive gurgling noise from your abdomen?
Leakage	Q34. Have you had any unintentional release (leakage) of wind or mucous?
Leakage	Q35. Have you had any unintentional release (leakage) of liquid stools?
Bowel Control	Q36. Have you needed to get up at night to open your bowels?
Bloating and Gas	Q37. Have you had abdominal pain or cramping not related to a bowel movement?
Pain	Q38. Have you had pain or cramping in your rectum (deep inside the back passage)?
Pain	Q39. Have you had pain /discomfort around your anal opening (back passage)?
Pain	Q40. Have you had bright blood in your stools?
Bowel Control	Q42. Have you been unable to wait 15 min to open your bowels?
Bowel Control	Q43. Have you had the feeling of being unable to completely empty your bowels?
Bowel Control	Q44. Does passing water cause your bowels to act immediately?
Emotional Function/Lifestyle	Q46. Have you had difficulty going out of the house, because you needed to be close to a toilet, because of bowel problems?
Emotional Function/Lifestyle	Q48. Did your treatment restrict the types of food you can eat due to your bowel problems?
Emotional Function/Lifestyle	Q49. Did you worry about your bowel problem?
Emotional Function/Lifestyle	Q50. Did you feel embarrassed by your bowel problem?
Emotional Function/Lifestyle	Q51. How unhappy would you feel if you lived the rest of your life with your bowel habit as it is now?
Single Item questions – not included in item scale testing	
Diarrhoea Medication (Single item)	Q52. Have you needed to take medication to control diarrhoea?
Bowel openings in 24 h (Single item)	Q53. What was the highest number of times you had to open your bowels in any 24 h period? Please indicate number in box
Requesting more assistance (Single item)	Q54. Would you like more assistance to manage your bowel problem? (optional question)

Multi-trait scaling analysis supported the EFA with one exception (Q46), which was moved out of the Bowel Control scale to the Emotional function/Lifestyle to correct for an indicated scaling failure. The results of the final multi-trait scaling analysis are in Table 4.

**Table 4:** Item scaling tests: convergent and discriminant validity for the QLQ PRT20 multi-item scales

QLQ PRT20 Scales	N items in scale	Convergent validity (range of correlations)	Discriminant validity (range of correlations)	Scaling successes^a^	Scaling success rate^b^	Homogeneity (average inter-item *r*)	Inter-item *r* > 0.3 & < 0.7 (%)	Within scale Reliability (Cronbach’s α)
Bowel Control	4	0. 48–0.59	0.31–0.52	16/16	100%	0.431	100%	0.749
Bloating and Gas	4	0.48–0.66	0.25–0.46	13/16	81%	0.453	100%	0.767
Emotional Function/Lifestyle	5	0.56–0.76	0.26–0.56	19/20	95%	0.526	90%	0.843
Pain	3	0.40–0.67	0.17–0.53	11/12	92%	0.476	100%	0.732
Leakage	2	0.53	0.25–0.43	8/8	100%	0.530	100%	0.693

^a^ Number of convergent correlations significantly higher than the discriminant correlations/total number of correlations, ^b^ Scaling success rate scaling success as a percentage

#### Reliability

Overall, the reliability of the scales were acceptable with Cronbach α’s exceeding 0.70 for Bowel Control, Bloating and Gas, and Emotional Function/Lifestyle scales (Table 4) [28]. The remaining scale; Leakage, was close to this level (0.693).

Test-retest reliability and validity analyses were performed on the linear transforms (outcomes 0–100) for the proposed scales similar to that outlined in the EORTC scoring manual [22]. Pre-treatment test–retest reliability (T1 and T2) results are presented in Table 5. The ICCs for each of the multi-item scales were rated as fair (> 0.6) to acceptable (> 0.7).

**Table 5:** Test – retest reliability from Time 1 (2 weeks before treatment) to Time 2 (week of treatment), ICC and Spearman’s Rho; multi-item scales linear transformed data, single item scales raw data

QLQ-PRT20 Scales [range], n	T1 Mean(*SD*) [range]	T2 Mean(*SD*) [range]	ICC of proposed scale** or kappa	T1 –T2 Spearman’s Rho or Phi
Bowel Control [0–100], 329	8.95(*15.43*) [0–83]	10.18(*15.60*) [0–100]	0.77	0.68*
Bloating and Gas [0–100], 329	6.35(*14.19*) [0–73]	7.58(*13.83*) [0–75]	0.61	0.67*
Emotional Function/Lifestyle [0–100], 328	9.55(*13.65*) [0–67]	11.56(*14.75*) [0–83]	0.74	0.62*
Pain [0–100], 329	5.17(*12.32*) [0–89]	5.51(*11.56*) [0–78]	0.76	0.64*
Leakage [0–100], 329	4.68(*12.77*) [0–100]	5.72(*13.14*) [0–100]	0.67	0.48*
Diarrhoea Medication^a^ [yes/no], 325	Yes = 8	Yes = 13	0.30 ^**k**^	0.31^P^*
Bowel openings in 24 h^a^ [0 +], 316	2.26(*2.23*) [0–25]	2.47(*1.96*) [1–20]	0.72	0.70*
Requesting more assistance^a^ [yes/no], 304	Yes = 33	Yes = 29	0.57 ^**k**^	0.57^P^*

^a^ Single item scales raw data, k = kappa, *p* = Phi, * *p* < .01, ** linear transformed data

#### Convergent validity

The QLQ-PRT20 scales correlated well with the predicted scales from the QLQ-C30 (Table 6). Notably, the QLQ-PRT20 Emotional Functioning/Lifestyle scale demonstrated relatively high correlations with all the selected scales of the QLQ-C30, and the QLQ-C30 Pain Symptom scale showed similarly high correlations with all the QLQ-PRT20 multi-item scales with the exception of Leakage.

**Table 6:** Pearson correlations between selected scales from the QLQ-C30 and the QLQ-PRT20 scales

Comparative scales	Bowel Control	Bloating and Gas	Emotional function/Lifestyle	Pain	Leakage
Global Health Status/QoL (QLQ-C30)	0.39	0.39	0.51*	0.35	0.28
Emotional Functioning (QLQ-C30)	0.26	0.27	0.42*	0.30	0.27
Social Functioning (QLQ-C30)	0.29	0.34	0.40*	0.29	0.17
Pain (QLQ-C30 Symptom scale)	0.42*	0.42*	0.47*	0.41*	0.25

* Correlations above 0.4, (all correlations were significant at *p* < .001)

#### Discriminant validity

##### Known-group comparisons

The QLQ-PRT20 scales were assessed on their ability to discriminate patient groups based on their age, cancer type (prostate, cervix or rectal), presence of co-morbidities, and RTOG scores (Table 7). All QLQ-PRT20 scales discriminated between patients showing acute symptoms of radiation proctitis and those without symptomology based on the T3 RTOG ratings (RTOG 0–no symptoms vs. RTOG 1 + acute symptoms; *p* ≤ .02, *r’s* = 0.16–0.37). Scores for Emotional Function/Lifestyle (*p* = 0.011, *r* = 0.17) and Leakage (*p* = 0.043, *r* = 0.14) further discriminated between mild or moderate symptoms. Examining the point estimates and 95% CI’s of the QLQ-PRT20 scales based on the absence or presence of RP symptoms (based on the RTOG scores) suggest that scores above 20 for the Bowel Control, Bloating and Gas, and Emotional Function and Lifestyle scales are indicative of significant concern (Additional file 3). For the Pain and Leakage scales, scores above 15 appear to be of significant concern.

**Table 7:** Known groups analyses: Median values, test statistics and *p* values for the known group’s analysis based on the T3 RTOG physician ratings, cancer type, upper and lower age quartiles and the presence of comorbidities

QLQ PRT20 Proposed Scale	T3 RTOG^a^: 0 (*n* = 108) vs. 1 (*n* = 153) vs. 2–3^c^ (*n* = 36)	Cancer Site^a^: Prostate (*n* = 264, male) vs. Cervix (*n* = 18, female) vs. Rectum (*n* = 28, M/F = 20/8)	Age quartiles^b^: lower ≤63 years (*n* = 88) vs. upper > 74 years (*n* = 95)	Presence of comorbidities^b^: No (*n* = 162) vs. Yes (*n* = 196).
Bowel Control	8.3 vs 16.7 vs 25: *H*(2) *=* 19.9, ***p*** **< .001**	–	–	–
Bloating and Gas	8.3 vs 16.7 vs 33.3: *H*(2) *=* 20.5*,* ***p*** **< .001**	16.7 vs 33.3 vs 16.7: *H*(2) = 38.3, ***p*** **< .001**	25.0 vs 13.9, *U* = 2252.5, ***p*** **= 0.007**	–
Emotional Function/Lifestyle	0.0 vs 20.0 vs 33.3: *H*(2) *= 51.4,* ***p*** **< .001**	6.7 vs 40.0 vs 36.7: *H*(2) = 12.8, ***p*** **= .002**	13.3 vs 6.7, *U* = 2177.5, ***p*** **= 0.003**	13.3 vs 10.0, *U* = 11,742.5, ***p*** **= .034**
Pain	0.0 vs 11.1 vs 16.7: *H*(2) *=* 20.5*,* ***p*** **< .001**	16.7 vs 33.3 vs 16.7: *H*(2) = 7.1, ***p*** **= .028**	–	–
Leakage	0.0 vs 0.0 vs 16.7: *H*(2) *=* 18.1*,* ***p*** **< .001**	–	–	–

^a^Independent samples Kruskal Wallis Test – *H*(degrees of freedom), ^b^ Independent samples Mann-Whitney U test, ^c^ T3 RTOG ratings above 2 were collapsed into the 2–3 group (there were no category 4 ratings)

*NB* Nonsignificant results not shown

Cancer type/location discriminated between acute and no symptomology for three of the scales; Bloating and Gas, Emotional Function/Lifestyle and Pain. Emotional Function/Lifestyle was the best discriminating scale with both cancers of the rectum and cervix showing significantly higher social/emotional disturbance ratings than the prostate group (*p* < .001, *r* = 0.28_rectal_ and *r* = 0.25_cervix_). The Bloating and Gas scale, reported higher ratings for the cervix compared to the prostate group (*p* = .004, *r =* 0.19). The Pain scale showed a trend for higher rating in cervix group compared to the prostate group (*p* = .074, *r =* 0.13).

Younger (≤=63 years) respondents reported higher levels of impact/symptoms for both the Emotional Functioning/Lifestyle and Bloating and Gas scales when compared to older (> 74 years) respondents (*r =* 0.24 and *r =* 0.22 respectively).

##### Responsiveness to change

The QLQ-PRT20 scales all demonstrated a strong ability to detect post treatment change in the patients from T2 (first week of treatment) to T3 (end of treatment): MeanΔ’s 7.5–12, all *p* < .001).

## Discussion

This study examined the reliability, validity and psychometric properties of the EORTC QLQ-PRT23 in an international sample of 358 patients receiving pelvic radiotherapy. Individual items and the internal structure of the scale were reviewed. The revised version, the QLQ-PRT20 comprises five dimensions: Bowel Control; Bloating and Gas; Emotional Function/Lifestyle; Pain; and Leakage. Three additional items are included to gain specific clinical information relevant to patient comfort and future treatment.

The proposed scales structure of the PRT20 have good internal reliability and face validity. Overall, the internal psychometric properties are strong. Eighty-six percent of participants completed the questionnaire within 15 minutes and participants did not find the questionnaire problematic/distressing.

The QLQ-PRT20 scales correlated well with the predicted scales from the QLQ-C30, suggesting good convergent validity. The QLQ-PRT20 Emotional Function/Lifestyle scale correlated well with all scales of the QLQ-30 which is appropriate as they are measuring similar things. Furthermore, the QLQ-C30 Pain Symptom scale and Global Health Status of the QLQ-C30 showed moderate-high correlations with all the QLQ-PRT20 multi-item scales apart from the Leakage scale. The fact that leakage did not correlate well with any of the QLQ-C30 items may be because the scales are measuring different lifestyle components and leakage is not likely to be a complaint unless patients are receiving pelvic treatment. The items within the leakage scale focus on unintentional release (leakage) of wind, mucous or liquid stools which are pertinent to proctitis. The leakage scale was kept separate to bowel control and bloating and gas because the questions focus on different symptoms and psychometric analyses suggested a better fit as an individual scale. The Emotional and Social Functioning on the QLQ-C30 also did not correlate well with bowel control and bloating and gas on the QLQ-PRT20; however, this is to be expected because the scales are measuring different lifestyle components.

The Emotional Function/Lifestyle scale discriminated for tumour type/location, age and presence of comorbidities. The Bloating and Gas scale discriminated between tumour type/location and age groups. This suggests that pain and leakage symptoms were similar regardless of age, tumour location and comorbidities, bowel issues did not vary based on age or comorbidities and bloating and gas symptoms were the same regardless of comorbidities.

The QLQ-PRT20 discriminated consistently between patients showing acute symptoms of radiation proctitis and those without symptomology based on the treating physician RTOG ratings. Scores on the QLQ-PRT20 Emotional Function/Lifestyle and Leakage scales further discriminated between those based on the degree of their symptomology (mild or moderate symptoms). The QLQ-PRT20 is suitable for measuring patient QoL because it provides more detail than using a clinician completed RTOG score, particularly when acknowledging weaknesses of clinician completed scales such as RTOG have been identified [29]. The QLQ-PRT20 also demonstrated a good ability to detect change in symptomology over time showing it to be appropriate for use in monitoring acute symptoms following pelvic radiotherapy.

Therefore, we contend that the QLQ-PRT20 alongside the EORTC QLQ-C30 is a suitable patient reported outcome that can be used to complement physician completed scales such as the RTOG scale. It provides a detailed picture of actual symptoms patients are experiencing following pelvic radiotherapy as well as providing a measure of how well the patient is coping with symptoms. The QLQ-PRT20 is different to site-specific modules because it focuses on quality of life related to side effects rather than treatment site and is effective in detecting symptoms specifically related to bowel issues experienced because of radiotherapy. We recommend that this module be used in conjunction with the EORTC QLQ-C30 to monitor patients’ side effects for pelvic radiotherapy.

A strength of this study is that it followed the EORTC QLG guidelines for module development for designing the study [19] and statistical analysis [22]. Limitations include recruitment of a larger proportion of prostate cancer patients and less female patients. The intention of the study was to get broad coverage across a range of representative diagnoses because the focus was on treatment-related QOL rather than diagnosis-specific. Although there were differences in the populations recruited between the countries (e.g. more females were recruited from Italy and more prostate cancer patients overall) the response patterns for the T3 QLQ-PRT23 (i.e., clinically relevant) were compared graphically and found to be generally similar overall across the language groups. A further limitation is that this study did not fully reflect the coverage of countries/languages recommended within the EORTC QLG guidelines. The module was tested in four languages with a large proportion of patients being recruited from Australia (187/358), which may have caused some biases in relation to language and culture. This was due to challenges in accessing and recruiting sites by the study team. Whilst this clearly limits the conclusions drawn on the cross-cultural applicability, the study did cover broader regions based on language (Australia (English); Norway, Canada/France and Italy) recommended with the exception of Eastern Europe. However, it could be argued that it is unlikely that the results would have been very different had more international participants been recruited. Testing of the QLQ-BIL21 similarly reported that they recruited a larger proportion of participants in one country [30]. Further research is needed to establish cross-cultural applicability of this module in different languages. Despite these limitations, we have shown that this module is relevant to patients in four countries with different diagnoses who received pelvic radiotherapy and demonstrated the scale structure, validity, reliability, and ability of this instrument to discriminate between symptoms.

## Conclusion

The EORTC QLQ-PRT20 alongside the EORTC QLQ-C30 module is quick and easy to complete, acceptable to patients, has good content validity and high reliability (including test-retest reliability). Furthermore, we have shown that it can discriminate symptoms from clinician completed scoring and for age, gender and comorbidities. Further studies are required to determine the incidence of proctitis for different tumour sites and treatment regimens.

## Additional files

Additional file 1: Treatment received by participants. (DOCX 18 kb)

Additional file 2: Number of patients with existing co-morbidities and type. (DOCX 13 kb)

Additional file 3: Means and 95% confidence intervals, range of the proposed QLQ PRT20 Proposed Scale compared to the RTOG score ratings indicting no symptoms (score = 0) vs. acute symptoms (score = 1+). (DOCX 13 kb)

## Abbreviations

ANZCTR: Australian and New Zealand Clinical Trials Registry
CX24: module for cervical cancer
EFA: Exploratory Factor Analysis
EORTC QLG: EORTC Quality of Life Group
EORTC: European Organisation for Research and Treatment of Cancer
ICC: Intra-class correlations coefficients () of the proposed scales between
PR25: module for prostate cancer
QLQ-C30: EORTC core questionnaire for quality of life
QLQ-PRT20: quality of life questionnaire for proctitis
QoL: quality of life
RTOG: Radiation Therapy Oncology Group scale
T1: Time 1 = two weeks before treatment
T2: Time 2 = first week of treatment
T3: Time 3 = post treatment
T4: Time 4 = At the three to six months scheduled follow-up appointment, after treatment completion
