# Supplementary material for: International validation of the EORTC QLQ-PRT20 module for assessment of quality of life symptoms relating to radiation proctitis: a phase IV study
Source: Radiat Oncol. 2018 Aug 29;13:162. doi: 10.1186/s13014-018-1107-x (PMC6116442; doi:10.1186/s13014-018-1107-x)
Supplement: Supplementary file 1 — Treatment received by participants. (DOCX 18 kb) [file 13014_2018_1107_MOESM1_ESM.docx]

Additional file 1: Treatment received by participants

| **Site/Dose*** | **Number of patients** | **Brachytherapy Boost and Dose^** | **Surgery at treatment site** | **Chemotherapy^#^** | **Progression /**  **Recurrence (at time of treatment)** |
| --- | --- | --- | --- | --- | --- |
| **Anal canal** | **3** |  |  | **1** |  |
| 54 Gy | 1 |  |  | 1 |  |
| 60 Gy | 2 |  |  |  |  |
| **Bladder** | **4** |  | **1** | **2** | **2** |
| 50 Gy | 1 |  | 1 |  | 1 |
| 66 Gy | 3 |  |  | 2 | 1 |
| **Cervix** | **19** | **15** | **16** | **13** | **1** |
| 30 - 37 Gy | 2 | 2 (15-21Gy) | 1 | 1 |  |
| 45 Gy | 12 | 12 (12-21 Gy) | 11 | 8 |  |
| 50 - 50.4 Gy | 4 | 1 (6 Gy) | 4 | 3 | 1 |
| 66 Gy | 1 |  |  | 1 |  |
| **Chordoma** | **1** |  | **1** |  |  |
| 50 Gy | 1 |  | 1 |  |  |
| **Endometrium** | **10** | **6** | **8** | **3** |  |
| 45 Gy | 5 | 5 (12-18 Gy) | 5 |  |  |
| 50.4 – 57 Gy | 3 | 1 (10 Gy) | 2 | 2 |  |
| 62.5 - 64 Gy | 2 |  | 1 | 1 |  |
| **Iliac nodes** | **1** |  | **1** | **1** |  |
| 50 Gy | 1 |  | 1 | 1 |  |
| **Prostate** | **286** | **3** | **77** | **1** | **17** |
| 45 Gy | 3 | 3 (19.5 Gy) |  |  |  |
| 55 - 69 Gy | 82 |  | 56 |  | 11 |
| 70 - 72.6 Gy | 22 |  | 10 |  | 3 |
| 74 - 76 Gy | 135 |  | 11 | 1 | 3 |
| 78 - 80 Gy | 44 |  |  |  |  |
| **Rectum^+^** | **28** |  | **25** | **26** | **2** |
| 45 Gy | 2 |  | 2 | 2 | 1 |
| 50 - 50.4 Gy | 14 |  | 13 | 14 |  |
| 54 Gy | 12 |  | 10 | 10 | 1 |
| **Sigmoid colon** | **2** |  | **1** |  | **1** |
| 45 Gy | 1 |  |  |  |  |
| 54 Gy | 1 |  | 1 |  | 1 |
| **Total number of patients** | **358** | **24** | **130** | **48** | **24** |

*1.8 - 2Gy per fraction was the standard dose per fraction used; ^ brachytherapy boost dose given in 1-3 fractions, 6-10 Gy per fraction. ^#^concurrent and/or sequential chemotherapy received, ^+^4 patients did not complete planned radiation treatment.
