# Supplementary material for: International validation of the EORTC QLQ-PRT20 module for assessment of quality of life symptoms relating to radiation proctitis: a phase IV study
Source: Radiat Oncol. 2018 Aug 29;13:162. doi: 10.1186/s13014-018-1107-x (PMC6116442; doi:10.1186/s13014-018-1107-x)
Supplement: Supplementary file 2 — Number of patients with existing co-morbidities and type. (DOCX 13 kb) [file 13014_2018_1107_MOESM2_ESM.docx]

Additional file 2: Number of patients with existing co-morbidities and type

| Diabetes | High Blood Pressure | Coronary Heart Disease | Stroke | Asthma | Chronic Obstructive Pulmonary Disease (COPD) | Previous Unrelated Cancer Diagnosis | Other |
| --- | --- | --- | --- | --- | --- | --- | --- |
| 24 | 68 | 41 | 6 | 4 | 11 | 22 | 127 |
