# Supplementary material for: International validation of the EORTC QLQ-PRT20 module for assessment of quality of life symptoms relating to radiation proctitis: a phase IV study
Source: Radiat Oncol. 2018 Aug 29;13:162. doi: 10.1186/s13014-018-1107-x (PMC6116442; doi:10.1186/s13014-018-1107-x)
Supplement: Supplementary file 3 — Means and 95% confidence intervals, range of the proposed QLQ PRT20 Proposed Scale compared to the RTOG score ratings indicting no symptoms (score = 0) vs. acute symptoms (score = 1+). (DOCX 13 kb) [file 13014_2018_1107_MOESM3_ESM.docx]

Additional file 3: Means and 95 percent confidence intervals, range of the proposed QLQ PRT20 Proposed Scale compared to the RTOG score ratings indicting no symptoms (score = 0) vs. acute symptoms (score = 1+)

| RTOG score | Bowel Control mean (95%CI)  [range] | Emotional Function /Lifestyle  mean (95%CI)  [range] | Bloating/gas  mean (95%CI)  [range] | Pain  mean (95%CI)  [range] | Leakage  mean (95%CI)  [range] |
| --- | --- | --- | --- | --- | --- |
| 0 | 14 (11-17)  [0 - 83] | 10 (7-13)  [0 - 80] | 15 (12-18)  [0 - 83] | 9 (6-12)  [0 - 56] | 8 (6-11)  [0 - 67] |
| 1+ | 25 (22-28)  [0 - 100] | 25 (22-29)  [0 - 100] | 25 (22 - 27)  [0 - 92] | 19 (16-23)  [0 - 100] | 15 (13-18)  [0 - 67] |
